# Prevalence of Harmful/Traditional Medication Use in Traumatic Eye Injury

**DOI:** 10.5539/gjhs.v5n4p55

**Published:** 2013-03-20

**Authors:** Kayode Olumide Ajite, Olufunmilayo Christianah Fadamiro

**Affiliations:** 1 Ekiti State University Teaching Hospital, Ado Ekiti, Nigeria

**Keywords:** ocular injury, visual impairment, inaccessibility, ophthalmic services

## Abstract

**Aim:**

Ocular trauma of varying aetiologies do occur frequently, however when different traditional/harmful substances are applied before presentation in the hospital, prognosis in terms of visual outcome following treatment may be worse than expected. This study is aimed at determining the prevalence of harmful/traditional eye medication practices among patients with traumatic eye injury in a tertiary institution.

**Study Design/Setting:**

It is a retrospective study of patients seen at the eye clinic of the Ekiti State University Teaching Hospital (a state government owned hospital), Ado Ekiti, from January to December 2009.

**Method:**

A review of case notes (medical records) of patients with history of ocular trauma both open and closed globe injury and who presented to the eye clinic of the Ekiti State University Teaching Hospital, Ado Ekiti from January to December 2009 was carried out. Demographic data of the patients, nature of the ocular trauma, substances applied to the eyes, visual acuity at presentation and after treatment were extracted. Frequencies and percentages were used in analysing the data.

**Result:**

A total of 1420 new patients attended the eye clinic during the study period (January to December 2009). Forty eight (3.4%) applied various substances into their eyes after sustaining ocular injury. Substances applied include Kerosene 25%, cassava water 20.8%, breast milk 12.5%, personal urine 10.8%, and cow urine 8.3%. Nearly half of the patients 23 (47.9%) presented with low vision and after treatment there was no visual improvement in almost all of them, 22 (45.8%). The period before presentation ranges between 1hr -2weeks post injury. However, the number of monocular blindness reduced from 8 (16.7%) to 5 (10.4%) after treatment.

**Conclusion:**

Kerosene and Cassava water were the common substances applied to the injured eye. The use of these harmful and traditional eye medications on injured eyes can reduce further the visual prognosis despite ophthalmic intervention.

## 1. Introduction

Physical or chemical injuries of the eye can be a serious threat to vision if not treated appropriately and in a timely fashion (Eze, Chuka-Okosa, & Uche, 2009). The most obvious presentation of ocular trauma is redness, pain and reduction in vision or loss of vision of the affected eyes. Patients with history of ocular trauma were more likely to use traditional eye medications (TEMs) (Eze et al., 2009). Common forms of TEM used that have been reported were human breast milk, leafy matter, castor oil, cassava water, kerosene, hen's blood and plants extract (Ayanniyi, 2009; Ukponmwan & Momoh, 2010).

Flying pieces of wood, metal, glass, and stone are notorious for causing much of the eye trauma (Eze et al., 2009). Blunt injury by fist, ball (cricket ball, lawn tennis ball), and other high speed flying objects can strike the eye resulting in various degree of injury. Road traffic accidents (RTAs) with head and facial trauma may also have eye injuries (Odebode, Ademola-Popoola, Ojo, & Ayanniyi, 2005), these are usually severe in nature with multiple lacerations, splinters of glasses embedded in tissues, orbital fractures, severe hematoma and penetrating open-globe injuries with prolapsed of eye contents (Eze et al., 2009; Onakpoya, Adeoye, Adeoti, & Ajite, 2010). Farmers while working usually sustains eye injuries from vegetative origin (Onakpoya et al., 2010). Artisans such as welders, carpenters and glass cutters may also be involved in the injury.

Traditional eye medications are substances, which are either naturally occurring or artificial applied to the eyes for achieving a therapeutic aim (Prajna, Pillai, Manimegalai, & Srinivasan, 1999). Most of the times it contains inherent ingredients that are toxic to the eyes. Due to some reasons such as inaccessibility to ophthalmic services, poverty and ignorance of consequences, some people after sustaining ocular injury engage in instillation of harmful and traditional eye medication (Ntim-Amponsah, Amoaku, & Ofosu-Amaah, 2005). Furthermore, there are reports that a large number of patients still use TEM before presentation to the hospital (Ukponmwan et al., 2010). Courtright et al. reported that 33.8% of patients with corneal disease in Malawi used TEM before presentation to a hospital. A previous study in Benin, Nigeria reported that 1.72% of patients seen at a hospital-based eye clinic over a 6-month period had ocular complications from the use of TEM. (Ukponmwan et al., 2010). These acts may invariably affect the visual outcome of the ocular injury. Various studies reported the use of TEMs and associated reduction in visual acuity but few reported TEMs use in ocular trauma (Yorston & Foster, 1994; Courtright, Lewallen, Kanjaloti, & Divala, 1994). This study is aimed at determining the prevalence of traditional eye medication practices among patients with traumatic eye injury in a tertiary institution.

## 2. Methods

A retrospective review of case notes (medical records) of patients with ocular trauma and history of prior instillation of traditional medications who presented to the eye clinic of Ekiti State University Teaching Hospital, Ado Ekiti, from January to December 2009 was carried out. Approval for the study was obtained from the Institution ethical board (IRB). Relevant patient's information including age, gender, occupation, nature of the ocular trauma, and substances applied to the eyes were extracted. Others were visual acuities at presentation and after the treatment. The visual acuity was measured with Snellens chart placed at 6 metres from the patient. All the patients had slit lamp biomicroscope examination of the anterior segment done by an Ophthalmologist. Posterior segment was examines with both direct and indirect Ophthalmoscopes and where necessary pupils were dilated with Tropicamide eye drops to view the fundus adequately. SPSS version 16 (SPSS Inc. Illinois, Chicago) was used in analysing the data, frequencies and percentages were used in presenting the result.

## 3. Results

A total of 1420 new patients attended the eye clinic of Ekiti State University Teaching Hospital, Ado Ekiti during the study period (January to December 2009). Forty eight (3.4%) applied various substances into their eyes after sustaining ocular injury. Table 1 shows age and gender distribution of the patients (male to female ratio, 1:1.2). Most of the patients 34 (70.8%) were in the 21-50years age range. The vocational distribution of the patients is shown in (Table 2). Farming (39.6%) and trading (16.7%) were the two most common. Table 3 shows the substances the patients applied to their injured eyes and both kerosene 28.2% and cassava water 16.9% were the two most commonly used. Almost half of the patients 23 (47.9%) applied more than one substances into the eyes.(Table 3) Nearly half of the patients 23 (47.9%) presented with visual impairment and there was no visual improvement in almost all of them, 22 (45.8%) after treatment. However, the number of monocular blindness reduced from 8 (16.7%) to 5 (10.4%) after treatment (Table 4).

**Table 1:** Age and gender distribution of patients

Age Group (Years)	Gender		Frequency (%)

	Male	Female	
< 20	1	2	3(6.3)
21-30	5	3	8(16.7)
31-40	4	7	11(22.9)
41-50	5	10	15(31.3)
51-60	2	3	5(10.4)
61-70	3	1	4(8.3)
71-80	2	0	2(4.1) 48(100.0)
TOTAL	22	26	48(100.0)

**Table 2:** Occupation of the patients

Occupation	Frequency	Percent (%)
Farmer	19	39.6
Student	8	16.7
Trader	8	16.7
Civil Servant	5	10.3
Artisan	4	8.3
Housewife	2	4.2
Unemployed	2	4.2
TOTAL	48	100

**Table 3:** Substances applied to the eyes before presentation

Substances	Frequency	Percent (%)
Kerosene	16	33.3
Cassava Water	10	20.8
Personal Urine	7	14.6
Cow Urine	4	8.3
Breast Milk	4	8.3
Battery Water	2	4.2
Brake Fluid	2	4.2
Traditional Concoction	2	4.2
Onion	1	2.1
TOTAL	48	100

* 23 Patients applied more than one substance into the eye

**Table 4:** Presenting and post treatment visual acuity (affected eyes)

Category	Vision	Presenting Va. Eyes (%)	Post Treatment Va. Eyes (%)
6/4 -6/18	Normal Vision	17 (35.4)	21 (43.8)
<6/18 - 6/60	Visual Impairment	13 (27.1)	15 (31.3)
<6/60 – 3/60	Severe Visual Impairment	10 (20.8)	7 (14.5)
<3/60-Nlp	Blindness	8 (16.7)	5 (10.4)
Total		48 (100)	48 (100)

** Npl- No light perception

*** Va-Visual Acuity

* Low Vision refers to both visual impairment and severe visual impairment

## 4. Discussion

The occurrences of ocular trauma are common (Onakpoya et al., 2010). Ocular trauma is a significant preventable public health problem being the leading cause of unilateral blindness globally (Onakpoya et al., 2010). Previous epidemiological studies on eye injuries have identified major risk factors, these include age, gender, socio-economic status and lifestyle (Onakpoya et al., 2010). Due to some reasons such as inaccessibility to ophthalmic services, poverty and ignorance of consequences, some people after sustaining ocular injury engaged in instillation of traditional eye medication (Eze et al., 2009). The resulting visual impairment or blindness is preventable when appropriate and prompt management is instituted.

In this study, 48 (3.4.0%) applied various traditional substances into their eyes after sustaining ocular injury. There was almost equal gender distribution (slight female preponderance) in TEM use; this is similar to report by Courtright and Lewallen (Courtright & Lewallen, 1994). However, this study found the use of traditional eye medication to be most common among the patients in the 21-50years age range. This is of concern because it is a ‘working age group’ (the productive age) that drives the economy of the society. Could it be the study group sustained ocular injuries during these activities and were too busy to immediately present in the hospital following ocular trauma but rather engage in the use of ‘available/accessible’ traditional medications?

Furthermore, in this study, the most frequently used TEMs was kerosene 28.2% this is similar to a study done in Eastern Nigeria (Eze et al., 2009), where the commonest TEMs used were chemical substances. These chemicals include kerosene, battery water and cassava water. However in a study conducted in India (Prajna et al., 1999), breast milk was the commonest TEMs used. In another study, Nwosu and Obidiozor (2011) pointed out that the use of herbs, roots, and their derivatives were the most common types of traditional eye medicine used by their patients. This suggests that people tend to instil various substances available to them after sustaining ocular trauma. Farmers, traders and students were the leading users of these traditional eye medications after sustaining ocular injuries. Previous studies have similarly reported farmers, traders and artisans as people who frequently use TEMs (Eze et al., 2009; Nwosu & Onyekwe, 2003). Visual outcome after ocular trauma may be reduced or remained the same depending largely on the severity of the trauma. However when various substances are applied, visual prognosis may be compromised further even after definitive ophthalmic interventions have been offered. In our study, majority of the patients presented with visual impairment 23 (47.9%) according to World health Organisation (WHO) staging. (WHO refers to visual impairment as Visual acuity of < 6/18- 6/60). The proportion of patients with visual impairment was reduced slightly to 22 (45.8%) after various appropriate treatment. The magnitude of monocular blindness (16.7%) at presentation also reduced to 5 (10.4%) after treatment. These findings are similar to reports by other researchers (Yorston, 1994; Nwosu et al., 2003). The little improvement in visual outcome observed in these patients are worth mentioning although further prospective study on this subject could have brought out the effect of other factors such as the duration of injury before application of traditional eye medication and duration of injury prior to presentation in the hospital on eventual visual prognosis. Generally, the visual outcome of ocular trauma depends majorly among other indices on presenting visual acuity. Therefore, additional insult such as instillation of traditional eye medication will adversely affect the visual prognosis. It is important to emphasize strengthening of primary eye care so as to reduce the prevalence of avoidable monocular blindness arising from application of TEMs after sustaining ocular trauma.

There is a need for a sustained public enlightenment to educate the populace on the effect of traditional eye medications. Like any other retrospective study, limitation of this study is the loss to follow up those patients with the use of TEMs after sustaining ocular trauma that could have been included in the study.

## 5. Conclusion

Kerosene and Cassava water were common substances applied to the injured eye. The use of these harmful and traditional eye medications on injured eyes can reduce further the visual prognosis despite ophthalmic intervention. There is a need for a sustained public enlightenment to educate on the effect of harmful and traditional eye medication.
